# Gout and Its Treatment

**Published:** 1903-06

**Authors:** M. A. Auerbach

**Affiliations:** New York City. 83 Madison Street


### Gout and its Treatment.

By M. A. AUERBACH, Ph. G., M. D., New York City.

GOUT (podagra) is an acute or chronic affection, due to an abnormal accumulation of uric acid in the blood and tissues, causing various symptoms, of which arthritis is the most distinctive and significant.

The history—with the exception of the classic work of Thomas Sydenham, "Tractatus de podagra et hydrope," published in 1683, and based upon his own case,—he having been himself a victim for thirty or forty years,—of our knowledge of gout is the history of the development of its pathology, which begins with Wolloston's discovery in 1797, that the deposits in the joints and vicinity in this disease are largely composed of uric acid. Previous to this time another view of the nature of gout was accepted, though without tangible foundation. In 1784, Cullen promulgated the nervous theory of gout, which was adopted from G. E. Stahl.

The tendency to gout is more frequently inherited, but is also acquired. Between 50 and 60 per cent. of all cases of gout can be traced to ancestry, parents or grandparents. More men are gouty than women, and it is the male line through which the tendency is most frequently transmitted. It is not usually manifested until after thirty-five or forty years of age, sometimes even later, but the signs which are almost sure to be present in gout may show themselves before the twelfth year. While over-eating, especially of meats, and intemperate drinking, associated with the luxurious habits which grow out of the possession of wealth, are the most frequent causes of acquired gout, these last, however, are by no means essential. Sir Dyce Duckworth's studies of gout in what is probably the richest field in the world, London, go to show that many of the peasantry in Ireland, among whom gout is almost unknown, become gouty after having lived for a time in London. On the other hand, not every person who inherits a tendency to gout becomes gouty, since the primary causes above mentioned may have been wanting. In others the tendency may be so great as not to require the favoring causes.

While alcohol is an acknowledged cause of gout, it has been observed that something depends on the form in which it is presented. Malt liquors, especially the "heavy" English ales and beers, strong in alcohol, are more active in the production of gout than the lighter beers consumed in Germany and this country. The same being true of the strong and sweet wines, of which port and sherry are the type, while pure whiskey is less harmful. The glycogenic substances in the malts and sweet wines are probably responsible for the source of acid fermentation products in the stomach, which, after absorption, reduce the alkalinity of the blood and impair the solvent power of the uric acid. An interesting exciting cause of gout is lead poisoning. This is met particularly in England, and especially in London, as pointed out by Sir Alfred Garrod in 1854. It is, however, rare in other parts of Great Britain and Ireland, and is growing less in London. For in 1870, according to Garrod, 33 per cent. of people who suffered from gout had been poisoned by lead. It may be, as suggested by Alexander Haig, that the effect of plumbism is to diminish the alkalinity of the blood. In this country the combination is comparatively rare.

It cannot be claimed that the pathology of gout is thoroughly established, although so many facts bearing on it are well determined. In this respect it resembles diabetes mellitus. It is commonly admitted that uric acid is in some way causative. Whether, however, the uric acid thus responsible is the result of the increased formation or diminished excretion, or both, is not so generally acknowledged. The ultimate result, however, is the same, whether it be from diminished excretion or increased formation, or both; there is an accumulation of uric acid in the blood, which is responsible, first, for certain premonitory symptoms of gout, and second, for certain local symptoms. The latter are of an inflammatory nature, and consist essentially in pain, swelling and redness of joints, preferably the smaller ones, and especially of the metatarsophalangeal articulations of the great toe; more frequently, perhaps, of the left great toe.

As to the relation of the uric acid compounds to the local inflammation, it is scarcely necessary to say that uric acid does not exist as such in the blood, even in pathological conditions. The normal urates, as originally shown by Bence Jones and confirmed by Sir William Roberts, are quadriurates. Such is the composition of the urate-sediments in urine. In the pathological state these are converted into the less soluble biurates, which make up the local deposits. It has all along been considered that these deposits are the direct cause of the gouty inflammations.

The most recent studies on this subject reaffirm the older view, that the mechanical theory offers a natural and complete explanation. The crystalline urates precipitated in the cartilaginous and fibrous structures of the joints necessarily act as foreign bodies; they excite irritation, clog the lymph-channels, exercise pressure on the tissue elements and impede their nutritive operations.

These effects sufficiently account for the inflammation, pain and swelling which ensue, and explain the remoter degenerative changes which follow.

Of modern authorities, Ebstein is the most eminent who ascribes the excess of uric acid in the blood to an abnormal formation of this substance in the body, in parts also which do not normally produce it, as bone, marrow and cartilage. The lymph and blood becoming surcharged, a balance is commonly restored by increased renal excretion or perhaps by decomposition of the uric acid. When, however, from any cause the lymph moves slowly, the so-called premonitory symptoms of gout occur—namely, a "tired feeling," vague pains, and the like. If an actual stasis occurs, the acute attack appears. This concentrated solution of uric acid acts like a chemical poison on certain tissues, giving rise to "necrobiotic changes," and when these reach a certain change characterised by an acid reaction, the uric acid is deposited in them as sodium biurate. If this stage is not reached, the local symptoms disappear and the joints return to their natural state. So much of Ebstein's view as makes a previously degenerated state of the tissues a necessary condition of the deposit of uric acid is commonly admitted by modern authorities as represented by Ord and Dyce Duckworth.

The nervous theory of gout was promulgated by Cullen in 1873, as a mixed neuro-humeral rather than purely neural. Of modern authors Sir Dyce Duckworth has taken the most pains to elaborate and establish the view that gout is a neuro-humeral disease, the sum of which may be said to be as follows:

That the diseased states recognised as of undoubted gouty nature are primarily dependent on a functional disorder of the nervous system, and that thus gout is a primary neurosis. That this, like others may be inherited or acquired, modified, or even repressed, and may be associated with other neuroses. A large part of the symptoms known as gouty are due to perverted relations of uric acid and sodium salts caused by the neurosis.

Persons subject to attacks of gout sometimes have premonitory symptoms suggesting the approach of an attack. These vary with the individual and are significant only in each case. There may be headache, neuralgia, anyone of the numerous symptoms of deranged digestion, irregularity of the heart's action, palpitation, high tension of pulse, depression of spirit, drowsiness, disposition to yawn, a "tired feeling"—in fact any symptom which the patient learns to associate with the attack. Attacks are apparently also brought on by anything which lowers the vitality of the patient. On the other hand, a supper with wine or a single glass of champagne will often produce an attack.

The first true symptom of the typical attack is articular pain, commonly in the great toe, at the metacarpophalangeal joint, and with its appearance the premonitory symptoms usually pass away. The pain is extreme, sharp shooting and sudden, often awakening in the middle of the night a patient who has gone to bed in apparent good health and least expecting an attack. With this pain are the swelling, heat and discoloration. Rarely the attack begins with a slight chill. On the other hand, there may be pain without heat, redness or swelling and all the typical local anatomical features of an attack without pain. In some instances the attack develops more slowly. At times the first attack is so slightly distinctive that it is assumed to be something much more trifling, as rheumatism or the result of some slight injury, while the personal peculiarities, natural or acquired, always more or less influence the symptoms. After the nocturnal outburst, the extreme pain diminishes as morning advances, but it may, and in fact is likely to repeat itself for five or six days, when the attack terminates.

Some fever usually accompanies the onset of acute gout. The temperature promptly rises to 100° F., but does not far exceed it, 102° F. being the usual maximum attained. As in other acute diseases the temperature arises in the evening. The attack terminates with desquamation of the epidermis over the inflamed joint.

Changes in the urine are almost distinctive. It is scanty, acid, high-colored and of high specific gravity. It deposits urates on standing and often contains a small quantity of albumin. It contains sometimes sugar in small or even decided quantity, but I am certain that at other times the reaction of uric acid on cupric oxide is mistaken for glucose. During, or preceding an attack the uric acid excreted may be diminished, later it may be increased, but as elsewhere stated, a relative increase in uric acid is often mistaken for an absolute increase in the twenty-four hours' amount. The probabilities are that these changes are inconstant.

A recognised symptom of acute gout, and sometimes the only one, is, pharyngitis, and now the term of "gouty sore throat" is one in common use. There seems no way of distinguishing it locally from other forms of sore throat in which there is no decided swelling.

Gout is said to be retrocedent or metastatic when it disappears suddenly from its external site and there are substituted for the external symptoms derangements of some internal organ, especially the heart or stomach, or brain or urinary bladder. In the first there appear cardiac symptoms of varying severity, including pain, shortness of breath and irregularity of the heart's action; in the second, gastrointestinal pain, a sinking sensation, vomiting or diarrhea, often associated with severe mental excitement or depression; in the third, meningeal symptoms, and in the fourth, cystitis. More rare events are gouty orchitis, parotitis and urticaria, or other fugitive skin affections. Metastasis is more prone to occur in atonic cases. Sudden death has supervened in some instances, but post-mortem lesions of a definite kind seem to be wanting, at least lesions which can be held responsible for symptoms.

As repeated attacks of gout occur and the patient grows older, certain changes take place—the joints deformed by tophaceous and other deposits, the lipping, the seal-fin hand, the renal and arterio-vascular changes, and the interstitial nephritis, and the like. The urine is now increased, lighter hued, contains albumin, and a few hyaline and granular casts.

The treatment of gout easily divides itself into two parts: first, that of paroxysm or acute attacks; second, that of gouty diathesis or tendency, which eventuates in the acute attack. Taking up the latter first, it is plain that if uric acid is its cause, whatever diminishes the amount of uric acid in the economy must tend to relieve gout. It is plain, also, that we may diminish uric acid in two ways: first, by confining the gouty person to such food as produces a minimum of uric acid; second, by administering such medicines as will promote its solution and elimination. The first of these constitutes, in the main, the dietetic treatment, and second the medicinal.

*The Dietetic Treatment.*—It consists essentially in the elimination, as far as possible, from the dietary of all nitrogenous or albuminous principles, being those whose complete combustion results in urea and incomplete in uric acid. I say as far as possible, for it is practically impossible to eliminate them altogether. The foods which are the type of this class should, however, be altogether omitted.

The typical foods permissible from the standpoint of composition are milk, butter, fruits and the succulent vegetables, except beans and oatmeal. To these oysters and lobster may be added, moderately; fish, except those containing a large amount of protein, and, where extreme rigidity is not required, poultry in moderate amount; but all butcher's meat should be strictly forbidden. It is usual, also, to interdict the use of carbohydrates (starches and sugars,) as well as the hydrocarbons (fats and oils).

There are, however, other sorts of ingesta, also entirely or almost free from nitrogen, acknowledged to be both a predisposing and exciting cause of gout—namely, malt liquors and wines. These are composed of water, alcohol, carbohydrates and a trace of mineral matters, but no nitrogen. It is not easy at first thought to understand why these substances should be harmful. Experience, however, shows that the stronger wines, such as port, Madeira and sherry, by their continued use are very likely to produce gout, while the lighter wines,—the clarets, hocks and Moselle wines,—if taken in moderation, rarely cause it. After these, stout, porter and strong ales induce gout. Even lager beer, which contains but 3 per cent. of alcohol, is capable of acting similarly, and I have known many men who have been forced to give up this beverage because of its effect. Cider and perry least of all beverages predispose to gout. On the other hand, distilled spirits, especially whiskey, are almost entirely without effect in producing gout. Why is this? Apparently, the amount of alcohol is not the measure of the effect, for whiskey, gin, brandy and rum all contain more alcohol than any of the wines alluded to. If reference is made to the wines most apt to produce gout, it will be found that they are those which contain a considerable quantity of both sugar and alcohol. Such are port, sherry, and Madeira, all of which contain more than 15 per cent. of alcohol and much sugar; also sweet champagnes, containing 11 per cent. of alcohol. On the other hand, some very sweet wines, as Tokay, Malaga, and the higher Sauternes, which contain much sugar, produce gout less rapidly. It would seem that those liquors which contain alcohol in combination with other substances, especially sugar, are potent gout producers, particularly where they excite indigestion, probably by restricting elimination, rather than producing more uric acid.

As to the acidity of alcoholic drinks, their influence is pretty clearly as exciting causes. In this way act the beers, in which both alcohol and sugar are present in small amounts, but which are highly acid. An explanation of this fact is less ready from the standpoint that the acute attack of gout is due to reabsorption of the deposited uric acid by an alkaline blood, than on the supposition that the attack is due to the irritating effect of uric acid deposited in the joints, because of the diminished alkalinity of the blood induced by the absorbed acid. Whatever be the explanation, few facts in the clinical history of gout are better established than that the ingestion of acid is an exciting cause of attacks.

A most valuable adjuvant to the dietetic treatment, are the natural mineral waters. The waters which have heretofore received almost universal approval, are the alkaline waters, although those possessing purgative properties also enjoy much reputation.

The actual mineral waters which have acquired the greatest reputation in the treatment of gout, are those of which sodium bicarbonate is the chief ingredient, to which the calcium bicarbonate is regarded a valuable adjuvant. Such are the alkaline waters of Vals and Vichy in France, Eviaules-Bains in Switzerland, Neueuahr and Fuchingen in Prussia, Coutrexville and Vittel in the Vosges, France, and Dax in France. Other waters possessed of reputation in the treatment of gout, in which the quantity of alkaline bicarbonate is smaller, owe it to their combined alkaline and aperient properties, chiefly due to sodium sulphate and magnesium sulphate, and belong to the second category of remedies for the treatment of gout. Such are the alkaline and saline waters of Carlsbad and Marienbad, in Bohemia, Kronthal, in Nassau, and Brides-les-Bains, in Savoy. Then there are saline waters represented by Baden-Baden, Ems, Homburg, Kissingen, Wiesbaden, and our own Saratoga waters. In saline waters we are considerably more fortunate in this country, the Saratoga waters furnishing all that can be desired. Finally, there are the bitter acidulated and purgative waters. Hunyadi Jaños, Friedrichshalle and Rokoczy, in Hungary,

Pölua, in Bohemia, and Rubinat, in Spain,—rarely resorted to in gout, but useful as eliminating agents. Among the weaker aperient waters are those of Bedford Springs, Pa., in this country.

The use of these mineral waters is especially indicated in a continuous manner between the attacks with a view to averting them. Especially useful are the thermal waters in the chronic arthritic complications, in which their internal use is combined with bathing. In this connection may be mentioned Carlsbad and Marienbad, where also the mud-baths are employed, Baden-Baden, Ems, Wiesbaden, Hammon R'Irha in Algeria, available in winter, Plombures in the Vosges, and Dax in France. Homburg and Kissingen are also resorted to for their baths, although the waters are cold.

The second category of remedies — the aperients — are decidedly useful in gout, both as eliminators and to prepare the way for the absorption and prompt action of the alkaline bicarbonates. They are not, however, used at the present day as freely as a century ago, and they are commonly reserved for the acute attack.

Hygienic measures are also of importance in the treatment of gout. The patient should bathe daily, using the cool bath in the morning or the warm in the evening on retiring, as experience may determine to be the best. The skin should be thoroughly rubbed with a rough towel and daily exercise should be practised, an open-air, out-door life being desirable whenever possible.

*The Medicinal Treatment of an Acute Attack of Gout.*—As a rule, the use of medicines is reserved for the acute attack. From the earliest history of the disease, practice has recognised two classes of remedies in the treatment of gout—namely, alkalies and purgatives, the object of both being to eliminate the offender, the first by producing soluble combinations which pass off readily by the kidneys, the second to carry it off by the bowels.

First, as to the alkalies and alkaline combinations. My experience places the (sidonal) quinate of piperazine easily at the top, and while it is not so rapid in its effect in relieving the pain of an acute attack of gout, it is nevertheless an invaluable remedy, exceeding all others. During an attack it should be given in doses of ten grains every hour and a half to two hours, dissolved in at least eight ounces of distilled water. Even larger doses may be given with advantage if borne by the patient. With relief to the acute symptoms, the dose should be reduced; but the remedy should not be discontinued, and between attacks smaller doses should be kept up for some time. These, however, may be substituted by the natural mineral waters.

Among the eliminating remedies is the time-honored colchicum, a drug which is of undoubted value in gout, but which, in my experience, must yield the palm to sidonal (quinate of piperazine). For a long time its action was inexplicable, and it came to be spoken of as a specific in gout, as quinine in chills and mercury in syphilis.

The aperients commonly used in gout are the salines, of which the magnesium sulphate is the favorite. Sodium sulphate is also used, and it is the constituent of the most actively purgative mineral waters, the Hunyadi Jaños, Rokoczy and Friedrichshalle mineral waters—already mentioned, now largely used instead. A favorite combination of the older physicians, was magnesium sulphate, two drachms; magnesium carbonate, a scruple; suspended in an ounce of cinnamon water, two or three times a day, until active purgation results.

Colocynth is also employed in gout, and advantage is taken of this fact in the preparation of the secret remedy, known as Laville's tincture, which is very largely used by the laity, and which undoubtedly has a very prompt effect in many cases of acute gout. The following has been published by the *Druggists' Circular*, October, 1889, as the composition of Laville's remedy, as determined by analysis:

| | | |
|---|---|---|
| Quinine | 5 | parts. |
| Cinchonine | 5 | " |
| Colocynthin | 2.5 | " |
| Lime Salts | 5 | " |
| Water | 82.5 | " |
| Alcohol | 100 | " |
| Port Wine | 800 | " |

For the relief of an acute attack of gout, leeches, blisters, and cold have all been discontinued of late years, not only because they are useless, but also because their use has been followed by fatal attacks of the so-called internal gout. Warmth and moisture do, however, have a mollifying effect, which is increased if the liquid preparations of opium be associated. Cocain, which might be expected to be useful, operates only through open surfaces. Should the latter be present, a 5 per cent. solution may be applied on lint.

It constantly happens that the pain in a paroxysm of gout is so severe that it is impossible to wait until the effect of the sidonal (quinate of piperazine) is secured, and a hypodermic injection of morphine is absolutely necessary to relieve the sufferings of the patient. I must remind the reader, however, that as many old subjects of gout have contracted kidneys, the use of morphine under these circumstances is attended with some danger and the drug should be used with great caution.

All pressure by boots on joints disposed to gout should be carefully avoided, as well as injuries, as such influences undoubtedly are predisposing causes. Muscular and mental fatigue are exciting causes of acute attacks and should be avoided by the gouty.

The true nature of a metastatic attack having been determined, it must be relieved symptomatically, while efforts to stimulate a true external attack may be made by hot mustard foot-baths, sinapisms, and the like. It has even been suggested that a pint of champagne may be advised, this being the wine most frequently responsible for acute attacks.

The following case histories will be of interest:

Case I.—Mrs. N. S., aged 22; married; three children; family history negative. Personal history: three years previous to her first visit to my office (March 23, 1901,) she was suddenly seized at night with severe pains in her right leg, especially involving the large toe. There were no premonitory symptoms. She had always led a very regular life, had never indulged too freely in alcoholic beverages, but usually took a glass of stout with her mid-day meal. These attacks had recurred at various intervals with more severity than the first one, also accompanied with redness and swelling of the joints.

Upon examination I found tophaceous deposits in both ears, deposits and partial ankylosis of various joints, especially that of the right wrist. On March 23 she came to me for treatment, feeling very heavy and tired. The case was readily diagnosticated as gout and she was immediately given sidonal, or quinate of piperazin, in 10 gr. doses, repeated 8 times a day, making a total of 80 grs. a day. On the night of March 24, she had rather a severe attack and sent for me on the morning of the 25th. She was then placed upon a regular diet and was directed to continue the powders of sidonal (quinate of piperazin). I saw her again on March 27 and 29, at which times she was able to be up and about, but the joints were still swollen and painful. On April 1, the swelling had greatly subsided and on the

5th had entirely disappeared. Her wrist was the only joint that troubled her. On the 10th she called for further treatment. I broke up the adhesions, keeping her on a special diet and continued the sidonal (quinate of piperazin) in 15 gr. doses, four times daily. I saw her again on April 12, 17 and 29; the wrist showing slight improvement. Further visits were made May 6, 13 and 31, June 7 and 29. I did not see her again until October 28. Up to that time she had had no recurrence of the gout, there was also much improvement in the condition of the wrist. She made further visits to my office on November 2, 11, 14, 18, December 6 and 13, at which times she was again enjoying full use of her wrist. I made another examination in January 1, 1902, and a last one on January 24, at which time the tophaceous deposits had almost entirely disappeared.

Case II.—Mr. Wm. F. Clark; single; mother living; father supposed to have died from gout; at any rate, had suffered from that disease for many years before his death, but no other members of the family had been subject to it.

Personal history: up to March 23, 1901, had enjoyed exceptionally good health. His troubles, however, began after participating in a dinner on the above date, at which a longer sitting than usual was indulged in. The 24th and 25th were spent in agony. The pains in the limbs were of a shooting character and as night drew on became unbearable. The joints were hot and swollen, but the traditional involvement of the great toe was absent. On the evening of the 25th, I was called to treat Mr. F. and came very near diagnosticating his case as one of articular rheumatism, but fortunately took his family history into consideration as well as the cause of the attack—namely, a debauch, for later developments showed it to be an atypical case of gout.

Mr. F. was placed upon sidonal (quinate of piperazin) in 10 gr. doses, from 8 to 10 times daily, making a total of from 80 to 100 grs. per day. After the first eight doses (80 grs.) there was a marked decrease of pain, redness and swelling, until on the 30th the attack and its effect had practically disappeared, but left my patient in an extremely uneasy frame of mind. On April 4 he called at my office desiring me to keep him under my care until I thought there was no further fear of a recurrence. I saw the gentleman again on April 7, 11, 15 and 22, making analysis of the urine at each time, but found nothing to cause alarm. In May I saw him but twice, the 6th and 31st, for the same purpose, with the same result. In June I saw him five times, 13, 17, 20, 24 and 27, with negative results. And lastly in July, several visits with equally negative results.

Although during the latter stage of the treatment the dose was reduced, a rigid diet was enforced with the addition of bland mineral waters as a beverage. In fact this is a routine practice in all my cases of gout. I have met the patient in question a number of times and he has never had another attack. He still continues upon the prescribed diet.

Case III.—Mr. H. O.; born in Spain; single; aged 47; family history not obtainable. Called at my office on the evening of March 25, feeling "queer"; complained of shooting pains, headache and impaired vision. Never had a previous attack. Upon examination nothing unusual could be found. The case was then set down as one of uremic origin. A sample of urine was taken and found to contain a large amount of uric acid. The gentleman gave a personal history of very high living; being employed by a wine house, his business required his indulgence in considerable quantities of heavy wines and especially champagnes. I advised entire abstinence from vinous or spirituous beverages and restricted his diet; advised the use of some good lithia water, about half a gallon a day with 10 gr. doses of sidonal (quinate of piperazin) three times daily. Early on the morning of April 1, I was summoned in great haste. Mr. O. had been suffering great pain throughout the night, which toward morning had become unbearable. Upon examination, I found the large toe of the right foot twice its normal size and extremely sensitive, the adjoining joints were inflamed and painful upon pressure. Upon inquiry I found that my patient had neglected to follow my advice and had dined well and late only the night before. The powders of sidonal (piperazin quinate) were increased to 10 grs. every hour and a half, dissolved in a goblet of water. The offending member was enveloped in cotton for warmth as well as protection from external pressure or contact. On the next day I found my case somewhat improved, the swelling had almost entirely subsided and likewise considerable of the pain. The powders were decreased to one every two hours. On April 3, I called and found patient sitting up, but nursing the affected member. An examination of his urine showed a marked decrease of uric acid and great improvement otherwise. I reduced the powders to one every three hours. Subsequent visits were made and on April 21, patient was discharged. Mr. O. was, however, kept under observation until October 25, 1901, without any recurring attack.

Case IV.—Mr. H. McC.; married. Family history: father and mother both living in Germany—aged 76, 54 respectively—and enjoyed good health. Two brothers and one sister, all living, and had good health. Personal history: aged 36; has enjoyed good health all his life up to October, 1901, when he was taken with an acute attack of indigestion which lasted about three weeks. Since that time he has been suffering with severe headaches and a strange feeling of an impending danger. On February 7, 1902, I was called to his home and found Mr. McC. suffering from acute pains in his back and lower limbs. Upon inspection I found the joints swollen and red, very tender upon pressure, the slightest movements eliciting severe pains. There was some rigidity at this time, although probably of a voluntary nature. The urine showed abnormal amounts of uric acid. Upon further investigation his tongue was found to be heavily furred and he complained of constipation, movements occurring every third or fourth day. His habits were not such as would suggest gout and in this case, which is not a true case of gout, I have been unable to trace its real origin, unless due to the constipation, which latter may have played an important part through autointoxication.

Mr. McC. was placed on laxatives, tonic stomachics, sidonal (quinate of piperazin) in doses of 8 grs., every three hours, given in a glass of vichy. He was ordered on light diet and was advised to eat fruits. On February 9, I called again and found that he felt somewhat better, the swelling and tenderness had greatly subsided; there was still some redness and pain on motion. On February 12, the more severe symptoms had entirely abated, hence the dose of sidonal was reduced to 10 grs., three times daily. On February 24, patient left city for a short trip south, feeling entirely well. Mr. McC. returned March 15th and has been under my observation ever since. He has had no recurrence of the symptoms.

83 Madison Street.

